# A broadened estimate of syntactic and lexical ability from the MB-CDI

**DOI:** 10.1017/S0305000921000283

**Published:** 2021-05-11

**Authors:** Trevor K.M. DAY, Jed T. ELISON

**Affiliations:** 1 Institute of Child Development, University of Minnesota, Minneapolis, MN, USA; 2 Department of Pediatrics, University of Minnesota, Minneapolis, MN, USA

**Keywords:** language acquisition, lexical development, syntactic development, toddlers

## Abstract

A critical question in the study of language development is to understand lexical and syntactic acquisition, which play different roles in speech to the extent it would be natural to surmise they are acquired differently. As measured through the comprehension and production of closed-class words, syntactic ability emerges at roughly the 400-word mark. However, a significant proportion of the developmental work uses a coarse combination of function and content words on the MacArthur-Bates Communicative Development Inventory (MB-CDI). Using the MB-CDI Wordbank database, we implemented a factor analytic approach to distinguish between lexical and syntactic development from the Words and Sentences (WS) form that involves both function words and the explicit categorizations. Although the Words and Gestures (WG) form did not share the factor structure, common WG/WS elements recapitulate the expected age-related changes. This parsing of the MB-CDI may prove simple, yet fruitful in subsequent investigation.

## Introduction

Language acquisition is characterized by a cascading and overlapping series of changes across different domains. Acquisition begins before birth, as phonetic perception begins in the womb (Moon, Lagercrantz & Kuhl, 2013). During the first year of life, phonetic perception begins to narrow (Kuhl, Conboy, Padden, Nelson & Pruitt, 2005; Kuhl, Stevens, Hayashi, Deguchi, Kiritani & Iverson, 2006), although this period is longer in learners of multiple languages (Kuhl, Tsao & Liu, 2003). By the end of their first year they are linking words to objects in their daily lives (Werker & Yeung, 2005). Next is a rapid explosion in lexical inventory, from approximately 10 words at 12 months to nearly 600 by 30 months (Dromi, 1987). At approximately 18 months, children begin combining these words into sentences that grow steadily more complex (Fenson, Marchman, Thal, Dale, Reznik & Bates, 2007), which requires the grammaticalization of the relation between referents. By about 5 years of age syntactic development is thought to be nearly adult-like (Gleason & Ratner, 2017). Syntax promotes further lexical development, and points to higher-level regularities and concepts within a language such as the past, the future, and causal relations.

The notion of function words and content words is one of the oldest distinctions in (Indo-European) linguistics. Some ways of formalizing the classes are as follows: (1) content words have specific or detailed lexical content, where function words do not; (2) content words select top-down (e.g., a verb selects certain direct objects), but function words are determined by their lexical complement; and (3) content words enter theta marking, and function words do not. However, the distinction is not perfect: for example, some prepositions carry both semantics and play a necessary grammatical role in an utterance (Corver & van Riemsdijk, 2013). In English, content words (nouns, adjectives, and most verbs) are mostly “open”-class, where new words can be derived and coined without restriction, whereas the function words (e.g., pronouns, interrogatives, and demonstratives) are “closed”-class, where new words are not easily added to each category. However, cross-linguistically, these classes are not perfectly synonymous, and there is wide variation in which syntactic categories are open or closed (e.g., Bhat, 2000), including adjectives (Dixon & Aikhenvald, 2004).

This distinction is recognized in early work involving the MacArthur-Bates Communicative Development Inventories (MB-CDI; Fenson et al., 2007). Early MB-CDI findings showed growth in closed-class words follows growth in open-class lexical terms, rather than happening simultaneously, with the use of function words – which we take to reflect use of these words in sentences and therefore representative of “syntax”– starting roughly at the 400-word mark (Bates, Marchman, Thal, Fenson, Dale, Reznick, Reilly & Hartung, 1994). Mean length of utterance (MLU; R. Brown, 1973), the average number of words or morphemes out of a set of a child’s utterances is a common estimate of syntactic ability. Cross-linguistic estimates of syntactic ability, as measured by MLU, are more strongly predicted by lexical inventory size than age (Bates & Goodman, 1999).

Many later studies of language acquisition, including its relation to other areas of development, or that use language as a predictor, often estimate language ability by relying on percentiles, count, or percentage of words understood or produced on a standardized inventory, often the MB-CDI. However, such analyses seem to ignore the differing roles function and content words play in a sentence.

The distinction between content and function words is evident as early as a child’s first birthday. Function words and morphemes are high-frequency, short, and often occur at the borders of prosodic units. By 16 to 24 months, infants can infer the role of function words based on their complements (i.e., pronouns signal verbs, and determiners nouns), see Christophe, Millotte, Bernal and Lidz (2008) for a review of early function word recognition. Other case-study evidence suggests that syntactic categories such as adjectives, nouns, noun phrases, prepositions, and prepositional phrase are understood by age 2:6 (Valian, 1986)

Based on the theoretical and experimental findings of the distinction between function and content words in English, we hypothesize that the acquisition of these classes will be dissociable in inventories of children’s speech, independently of metrics such as MLU. In this study, we use both forms of the MB-CDI: Words & Gestures (WG; 8–18 months) and Words & Sentences (WS; 16–30 months). Although some authors have suggested that “syntax” may start as early as the two-word stage, when children are approximately between 18 and 24 months of age (Hyams & Orfitelli, 2015), WG provides no measures of syntactic ability in the vein of the WS sections. The goal of the present study is to examine (1) whether estimates of skill with content and function words separately can be generated from the complete WS form, informed by the additional morphological and syntactic ratings; and (2) whether this analysis is extendable to WG, despite (appropriately) lacking categories akin to WS “Sentences and Grammar”. Thus, the primary objective of this paper is to use an exploratory/confirmatory factor analysis (EFA/CFA) approach (e.g., T. A. Brown, 2015) to delineate latent categories on the MB-CDI and map them onto development of content and function vocabularies.

The repository of data we use for this study, the Stanford Wordbank (Frank, Braginsky, Yurovsky & Marchman, 2017) provides some demographic information from its constituent studies, including age, sex, mother’s education, birth order, and ethnicity. Long-standing findings hold that boys speak fewer words at a given age than girls (Bates et al., 1994; Bouchard, Trudeau, Sutton, Boudreault & Deneault, 2009; Van Hulle, Goldsmith & Lemery, 2004), and children of more highly educated mothers (Bates et al., 1994) – with mixed findings with respect to fathers (Bates et al., 1994; Pancsofar & Vernon-Feagans, 2006) – speak more words. Furthermore, first-born children often perform better on simple measures of language production and comprehension compared to later-born children (Bates et al., 1994; Berglund, Eriksson & Westerlund, 2005; Hoff-Ginsberg, 1998). We will perform exploratory analyses to see whether these demographic variables are differentially associated with lexical and syntactic ability, measured through content and function words, respectively.

## Methods

### Participants

The American English scores from 7,955 children between the ages of 10 and 30 months were obtained from Wordbank on November 5, 2019. All forms were included to capture the widest range of variability. Although the norming sample is the most strictly curated, the complete sample is not meant to include any children with developmental disorders. Data from 2,435 English WG forms and 5,520 WS forms were analyzed. Table 1 shows the summary data for demographic variables contained within Wordbank. For numeric analyses, mother’s education was converted to a numeric approximation based on typical North American education schemes (primary: 5; some secondary: 8; secondary: 12; some college: 14; college: 16; some graduate: 18; graduate: 20), rather than as a categorical variable.

### Instruments

WG provides an inventory of 396 words over 19 categories where caregivers are asked to rate whether their child “understands” or “says and understands” each word. WS provides an inventory of 680 words over 22 categories where caregivers are asked to rate whether their child produces each word. All words listed in WG are present in WS. WG “Actions and Gestures” (II), Actions and Gestures, which asks the rater whether the child performs certain gestures or actions such as “waving goodbye”, is not included as this article focuses on the word categories for comparability between WS and WG.

WS also contains a second part, “Sentences and Grammar”, designed to assess morphological and syntactic ability. It contains the five sections listed below in which raters are asked about a child’s language, where raters are asked:

1. Whether their child applies common English morphosyntactic rules. This section is excluded from this paper because it is short (five items), and the underlying forms also reflected in “Word Endings”.
2. **Word Forms:** To identify from a list which correct irregular noun (n = 5; e.g., *children*) and verb forms (n = 20; e.g., *ate*) their child produces.
3. **Word Endings:** Incorrect overgeneralization of morphological rules for nouns (n = 14; e.g., *childs) and verbs (n = 17; e.g., *ated) their child produces.
4. To report three of the longest sentences their child has said recently, which is used to compute MLU. This section was not included in the Wordbank data.
5. **Complexity:** To mark one sentence each out of 37 pairs of sentences, indicating what complexity level is most like their child’s speech (e.g., “Doggie kiss me” vs. “Doggie kissed me”).

### Analytic strategy

Factor analyses were performed using the *psych* package (version 2.0.9) (Revelle, 2018) in R (version 4.0.2, “Taking Off Again”) (R Core Team, 2020). WG lexical subsections were scored as 1 for “says and understands” and 0 for “understands” for consistency with WS. For lexical and morphological lists, each category was scored as proportion of words selected, and therefore given equal weight in analyses, as is necessary for factor analyses. WS sentence complexity was scored as proportion where the more complex option was chosen.

Before filling out WS “Complexity” (II.E), responders are asked if their child has begun combining words. If they choose “not yet”, they are not asked to fill out the sentence complexity section. These individuals (n = 1,426) were given a complexity score of 0%.

Factor analyses were performed by treating WG “Phrases” (I.B) categories as indicators; and WS “Vocabulary Checklist” I.A categories, as well as “Word Forms” (II.B), “Word Endings” (II.C), and “Complexity” (II.E), all equally weighted once scored as proportions endorsed. The categories and the number of items in each are given in Table 3.

### Words & Sentences analysis

**Figure 1.**
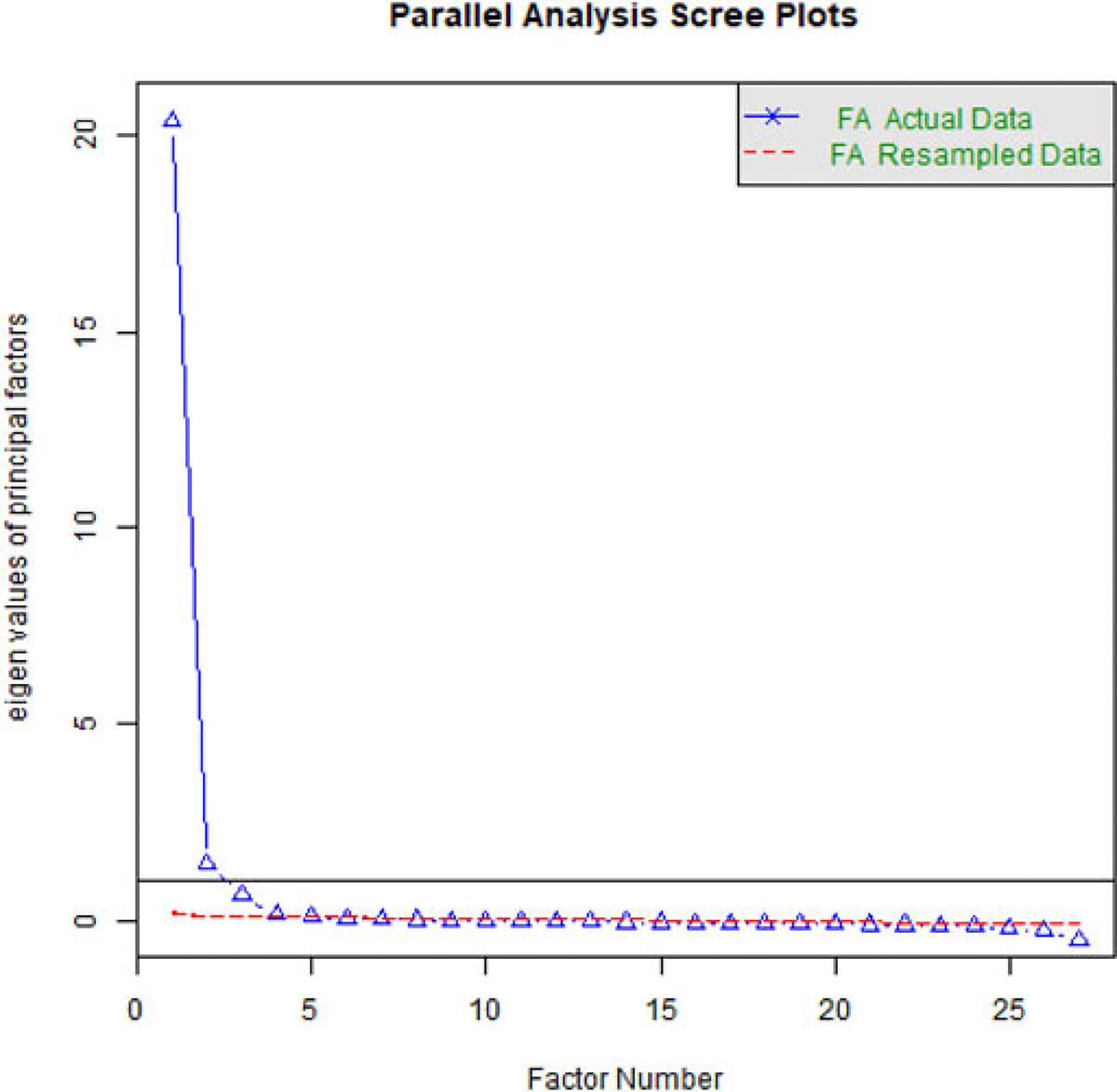
Scree plot for a principal axis factoring of the WS data, used to select the number of factors to extract from a subsequent FA. Two factors show the most improvement in variance explained. The dashed red line shows the eigenvalue for simulated random data; factor solutions below this line are considered to not be valid. The line at y = 1 is the Kaiser criterion for selecting factors; however, we also examine the third factor (just below).

A principle axis factoring (PAF) analysis of the EFA data (*n* = 2250) is used to guide factor number (*m*) selection for the EFAs. PAF identifies commonalities in the correlations between measurements (here, MB-CDI subsections). We use PAF to identify the most parsimonious model that explains the most variance. Figure 1 shows the parallel analysis scree plot of the eigenvalues for this PAF. The dashed redline shows simulated data with population correlations of 0. Factors with values below this line do not offer useful improvements on the model. The first value following a large drop is typically considered the most parsimonious fit; as subsequent smaller decreases in eigenvalue show decreasing improvement and interpretability on the model (Woods & Edwards, 2011). However, absolutist decisions about *m* are disfavored, so we explore *m* between these two cutoffs, at *m* = {2, 3}, using the oblique rotation method “oblimin” (Carroll, 1957), as lexical/syntactic ability are theoretically highly related. Oblique rotation does not force factors to be as orthogonal as possible. Each factor analysis (FA) was repeated 1,000 times to bootstrap confidence intervals. It is these results that are used for interpretation.

Data were split in half using stratified sampling with the *slice_sample()* function from the *tidyverse* package (ver. 1.3.0, Wickham et al., 2019), holding the most important potential predictors constant between halves: age, sex, mother’s education (including missing). An EFA was conducted on one half (n = 2250), and a CFA (n = 2270) on the other. Descriptive statistics for each group are included in Table 2.

### Words & Gestures analysis

The data from WG participants were similarly split into EFA (***n*** = 1214) and CFA (***n*** = 1221) halves. Descriptive statistics for each group are included in Table 2.

Following PAF guidance, EFAs were tested for WG at *m* = {2, 3}, using the same rotation method and iterations. Inventory scores were calculated as percent marked “says and understands” to maintain consistency with WS. Part II Actions and Gestures was not included.

### Joint analyses

While all words in the WG inventory exist in the WS inventory, they are not consistently categorized between forms, e.g., “church” is listed under WG “Outside Things” but WS “Places”; and “wait” under WG “Games and Routines” but WS “Action Words”. Therefore, WG words were redistributed as necessary according to the WS organization. Following redistribution, only the WS category “Connecting Words” had no items represented on WG. Furthermore, “in” and “inside” are separate items on WG but given as “in/inside” on WS. As a resolution, “in” was dropped from WG and “inside” was treated as corresponding to WS “in/inside”. “Connecting Words”, as well as morphology and syntax sections for WG, were modeled having scores of 0%.

Using the best factor solution, we then calculate the lexical and structural scores for all (*n* = 7, 955) individuals across the 10 – 30 month age range, regressing them against the demographic variables available in Wordbank. All code is available on GitHub (https://github.com/TrevorKMDay/MCDI-analysis).

## Results

### Words & Sentences

#### Factor structure

**Figure 2.**
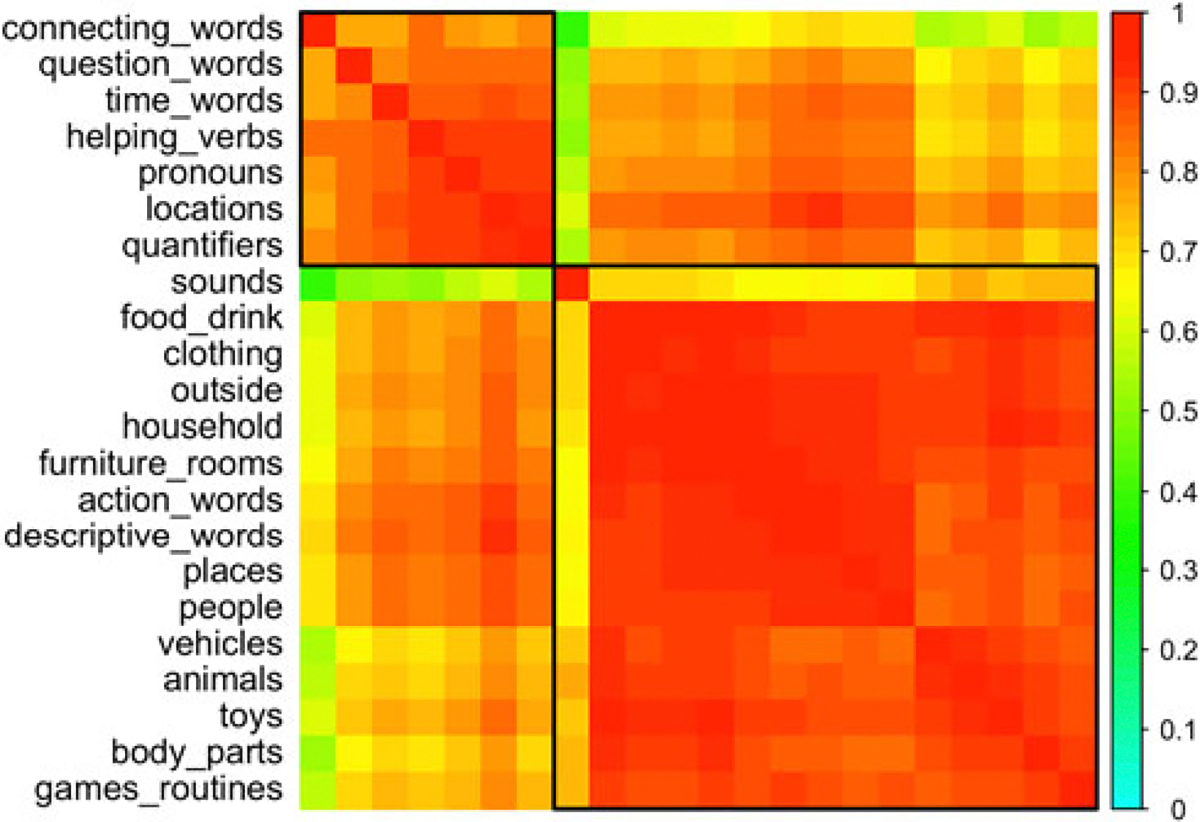
Correlations between lexical subcategories (WS). Note that all correlations are positive.

Postulating that words can be grouped into open-class lexical items and closed-class function words, a simple correlation of all lexical categories shows reasonable groupings (hclust) of (1) the final seven categories (*Words about Time, Pronouns, Question Words, Prepositions and Locations, Quantifiers and Articles, Helping Verbs,* and *Connecting Words*), hereafter “L7” (Figure 2), and (2) the remaining categories, hereafter “L15” (*Sound Effects and Animal Sounds, Animals, Vehicles, Toys, Food and Drink, Clothing, Body Parts, Small Household Items, Furniture and Rooms, Outside Things, Places to Go, People, Games and Routines, Action Words,* and *Descriptive Words*). All of L15 can be considered open-class. Of L7, all categories except *Words about Time* can easily be considered closed-class. That section (*after, before, day, later, morning, night, now, time, today, tomorrow, tonight, yesterday*) may share properties with both closed- and open-class words. See Table 4.

A priori, one might hypothesize successive factor solutions of the MB-CDI to represent (1) a general language factor; (2) lexical/structural ability; (3) lexical/syntactic/morphological ability. The utility of the four-factor division is not immediately obvious. Here, we discuss the two- and three-factor solutions as they are the most useful and interpretable. Although problematic in orthogonal FAs, loadings greater than 1 are permissible in oblique FAs.

The two-factor solution shows a clear division in factor assignments. There is no consensus at what loading value an indicator can be considered assigned. The cut-off we use, 0.4, is somewhat conservative (T. A. Brown, 2015). At this cut-off no indicators are cross-loaded. We see a clear division between the L15 lexical categories, and the L7 lexical categories and the four morphological categories (word endings, word forms) and the *Complexity* section. It is thus reasonable to name the former factor “lexical”, and the latter “structural”. The factor loadings are given in Table 4, where loadings ⩾ 0.4 are bolded. The correlation between the factor scores was .79, 95% CI [.77, .80].

The L15, “lexical” category is maintained: however, the structural factor is divided in two, with the L7 category joined by *Word Forms, Verbs* and *Complexity*, and the remaining morphological categories on their own in the third factor. However, at the same cut-off at 0.4, *Word Endings, Verbs* is cross-loaded between the latter categories. All indicators are assigned to a factor. The factor loadings are given in Table 4, where loadings ⩾ 0.4 are bolded. The correlations between the factors was: lexical/syntactic: .77, 95% CI [.75, .78]; lexical/morphological: .47 [.39, .57]; syntactic/morphological: .52 [.44, .60].

#### CFA

We used the *cfa* function from the package lavaan (Rosseel, 2012 ver. 0.6–6) to calculate model fit indices on the confirmatory half (*n* = 2270). Given the non-normality and non-independence of the indicators, using maximum likelihood parameter estimates (MLR), the CFA produced reasonable fit for the 2-factor solution. The model fit was evaluated using the following fit indices: model Chi-square, comparative fit index (CFI), the Tucker-Lewis index (TLI), root-mean-square error of approximation (RSMEA), and standardized root mean square residual (SRMR). However, we note Chi-square significance tests are almost always significant (indicating bad fit) in large sample sizes (Hooper, Coughlan & Mullen, 2008). The fit statistics for the two- and three-factor solutions are given in Table 6.

#### Theoretical validation

**Figure 3.**
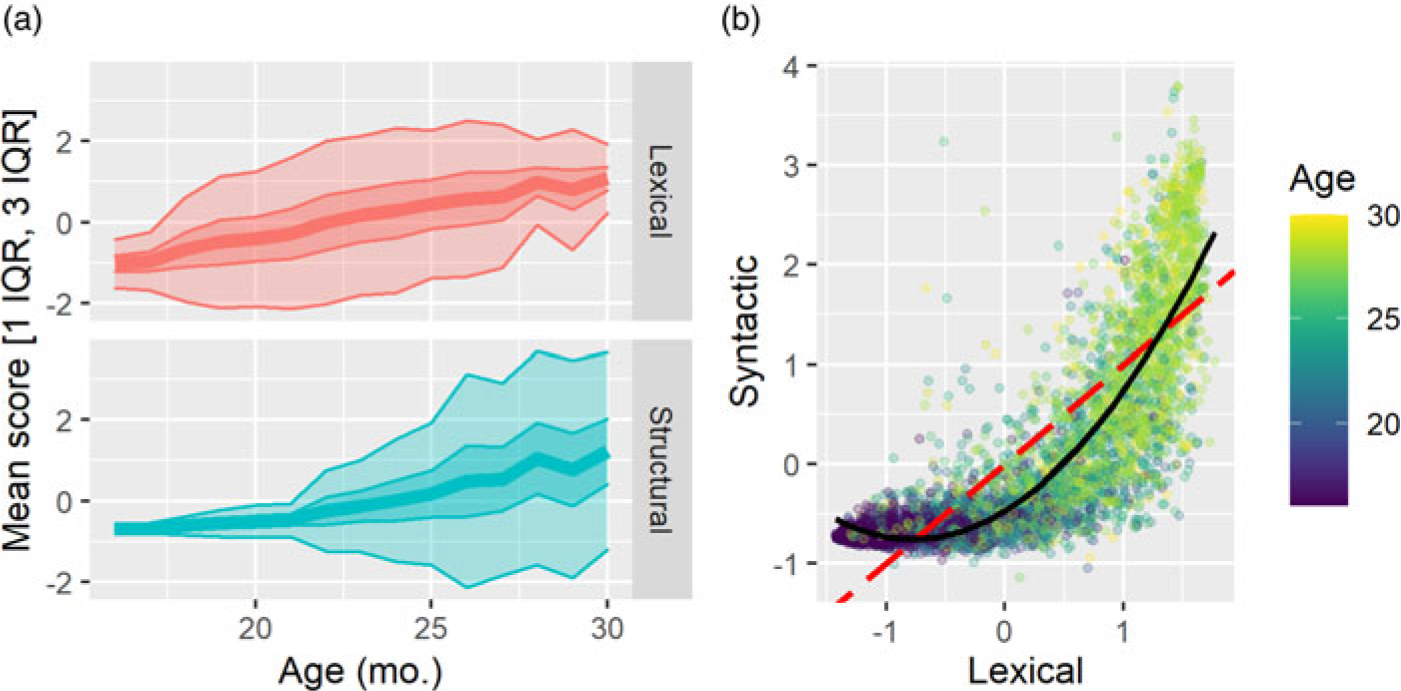
(a) Distribution of lexical/syntactic ability scores plotted by age band. The central line represents the mean score, and the bands 1 IQR (inner; 50%) and 3 IQR (outer; 99%). (b) Lexical and syntactic ability scores plotted against each other within-individual. The red dashed line indicates the hypothetical equal-development rate, and the solid black line indicates the second-degree polynomial line of best fit. Age is plotted youngest (purple) to oldest (yellow-green) using viridis (Garnier, 2018).

One test of the validity of our lexical/syntactic ability scores is whether syntactic ability lags lexical ability as measured in this framework. Figure 3 presents two arguments for this finding, using factor scores estimated over all 5,520 WS individuals (*psych* function *factor.scores*, estimation method *tenBerge*). Figure 3(a) shows that both lexical and syntactic ability scores increase over 16 to 30 months, with the variation in lexical ability narrowest (representing low variability) at younger age/low ability and higher age/higher ability (representing consolidation of ability across individuals). However, syntactic ability only begins to grow roughly in the 20- to 24-month age range. Variability is high and ability does not consolidate by 30 months.

Figure 3(b) shows a distinct lag effect, where normalized syntactic ability is almost always lower than normalized lexical ability, and syntactic ability only develops when lexical ability is highest. Perfectly synchronous development would result in a distribution surrounding the line *y* = 1, plotted in red. A second-degree polynomial regression produces a good fit, *R*^2^ = .760, *F*(2, 5517) = 8788, *p* < .001, 95% CI [0.749, 0.771].

#### Factor estimates

**Figure 4.**
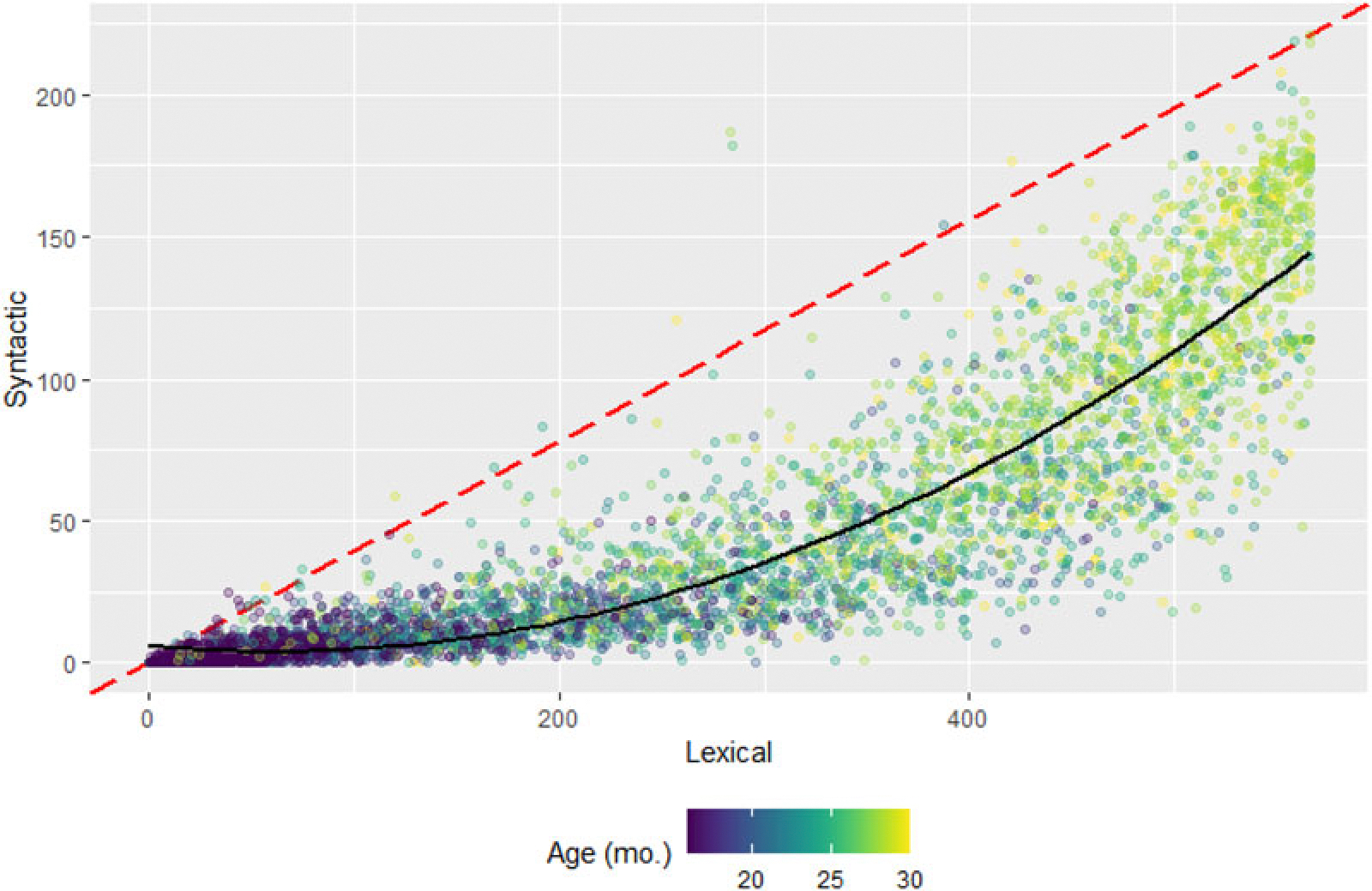
Estimates of lexical and syntactic ability scores plotted against each other. Each dot represents the scores from one individual. The red dashed line indicates the hypothetical equal-development rate, and the solid black line indicates the second-degree polynomial line of best fit. All 5520 WS individuals are included.

Presuming comprehensive characterizations of measurement invariance across various potential moderators, the underlying latent factor structure should hold across samples. An alternative approach would derive sums of items, but as we demonstrate, this approach yields highly colinear factors. Summed lexical and structural scores are the sum of the number of items endorsed across L15 and L7 for each individual. As a simple sum, this is easily done for any MB-CDI form and is comparable between studies (see Figure 4). However, the R^2^ between the lexical and syntactic scores in this formulation was .894, 95% CI [.889, .899], higher than the FA score R^2^ of .76 95% CI [0.749, 0.771], showing greater collinearity among the factor scores in the simple sum formulation. While FA scores should be used as a predictor or outcome; the summed approach has the benefits of (1) being applicable in samples too small for a FA; (2) being applicable in samples with theoretically different factor structure, and (3) being more interpretable.

### Words & Gestures

#### EFA

A similar EFA is significantly less clear for WG. At a cutoff of 0.4, the two-factor analysis groups *Words about Time, Action Words, Descriptive Words, Question Words, Quantifiers and Articles,* and *Pronouns,* but also *Furniture/Rooms*. Furthermore, *Household* and *Outside are* cross-loaded. A three-factor solution groups in one factor Q*uestion Words* and *Time Words,* but *Pronouns* is grouped with *Sound Effects and Animal Sounds, Animal Names, Toys, People,* and *Games and Routines*. The rest of the lexical categories are joined by *Prepositions and Locations* and *Quantifiers.* All indicators were assigned to a factor. The interpretation of these factors is unclear (Table 5).

The poor factor structure may be due to the relatively low subcategory scores. Across all subjects, only 8% of subcategory scores exceeded 30% endorsed. Even among the 18-month-olds, only 27% of subcategory scores exceeded 30% endorsed. This suggests that in typically developing toddlers between 8–18 months, a lexical/functional distinction is either not developed or not able to be estimated with WG.

#### CFA

WG CFAs were performed in the same manner on the confirmatory half (*n* = 1221). The two- and three-factor CFAs did not converge.

### Joint analysis

#### Factor structure

**Figure 5.**
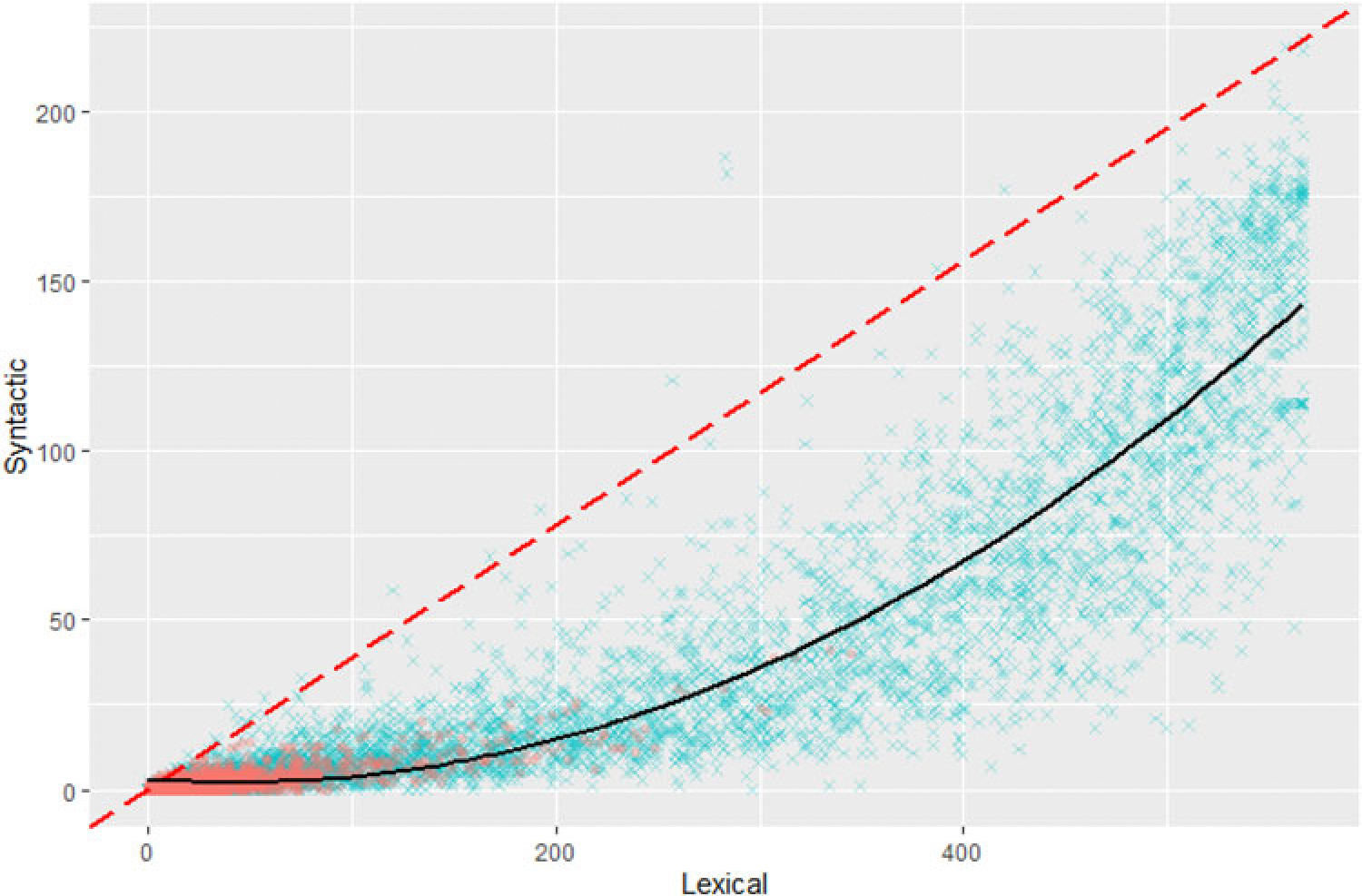
Distribution of lexical/syntactic ability scores plotted against one another within individual, in both WS (blue) and WG (red). Ages are obscured for clarity. The red dashed line represents y = 1, the hypothesized relationship if abilities developed at the same rate. All WG and WG individuals included.

We estimated factor scores (*psych* function *factor.scores*) using the WS-only structure as applied to all WS and WG participants jointly. Such an analysis creates a very similar distribution (Figure 5). While most WG individuals are grouped at the low end of both syntactic and lexical ability (lower left corner), notably, some individuals estimated to have greater syntactic and lexical ability than many WS individuals. A second-degree polynomial regression produces a good fit, *R*^2^ = .73, *F*(2, 7952) = 10930, *p* < .001.

#### Factor estimates

A similar linear combination of categories assigned to each group was performed; however, in this model, lexical ability explains more of the variance in syntactic ability compared to factor scores, *R*^2^ = .86, *F*(2, 7952) = 25090, *p* < .001, reducing the independence of the two score estimates.

#### Demographics

In the linear-estimate joint dataset, both lexical and syntactic ability are associated with age (lexical: *R*^2^ = .66, *F*(1, 7953) = 15500, *p* < .001; syntactic: *R*^2^ = .50, *F*(1, 7953) = 7971, *p* < .001). These estimates are used for interpretability of betas in the following section. For context, the lexical category comprises 566 items and the syntactic, 221.

We analyzed the effect of sex and mother’s education in a single model for each outcome, controlling for age, on lexical and syntactic scores (*n* = 3841, accounting for missing sex and mother’s education). Males had fewer words in both categories (lexical: β = −31.6 (5.5%), *p* < .001; syntactic: *β* = −9.2 (4.2%), *p* < .001). Mother’s education level affected lexical (*β* = 2.0 (0.35%), *p* < .001) and syntactic (*n* = 3842, *β* = .39 (0.18%), *p* = .0014) inventory size. Uncorrected *p* values are listed. However, these confounds are not regressed out in order to examine the full range of lexical/syntactic learning relationships.

Finally, in order to examine the possibility of a demographic-based differences in syntactic ability compared to lexical ability, we regressed syntactic score against lexical score squared (LEX:*β* = −0.073, *p* < .001; LEX^2^:*β* = 0.00054, *p* < .001) and age squared (age:*β* = −1.2, *p* < .001; age^2^:*β* = 0.059, *p* < .001), including sex and mother’s education. There was a significant effect of sex (*β* = −2.6, *p* < .001), but not mother’s education (*p* = .37). This effect was further tested by examining the interaction of sex with all four terms. In this analysis, sex (*β* = −12.7, *p* = .013) and the interactions with lexical score (sex*LEX:*β* = 0.0035, *p* = .73, sex*LEX^2^:*β* = −3.2 × 10^−7^, *p* = .99) were not significant.

## Discussion

The evidence suggests separate learning streams for lexical and syntactic ability, measured through content and function word production. While these categories are largely open- and closed-class, respectively, this analysis doesn’t distinguish whether the difference in acquisition timing is related to one categorization or the other. The two- and three-factor solutions had similar fit statistics, and based on their structure and parsimony, we propose that the two-factor solution is the most informative. The most important finding in this paper is that the categories nominally included in the lexical inventory on WS, together with the later morphological and syntactic items represent growth on an underlying factor, here termed “structural”, representing both developing knowledge of sentential structure and inflectional morphology. Follow-up research on English and similar languages should investigate the timing and relationship of derivational morphology to these factors, as correct derivation would indicate structural learning of syntactic categories and the adoption of language-specific derivational rules.

The morphological categories were left off the WG form entirely; due to the fact most children by 16 months are producing very few, if any, of the words indicated. However, some function words are included in the inventory section. While the FA was not successful, in part because of low endorsement rates, a longitudinal analysis might clarify the relationship between WG scores by involving more detailed WS scores through an analysis such as moderated nonlinear factor analysis (Bauer, 2017).

Another drawback is that both collect many more content items than function items: however, this is more a drawback to studying these features. However, the results of the FA mathematically support the theoretical grouping of categories. Although lexical and syntactic ability were highly correlated, syntactic ability was found to lag lexical ability throughout development, both between and within subjects.

Our findings of girls and children of more educated mothers knowing more words in both factors is consistent with long-standing and cross-linguistic findings (Bates et al., 1994; Bouchard et al., 2009; Van Hulle et al., 2004). Sex-based differences, but not mother’s education, were evident in examining differences in syntax, controlling for lexical inventory size.

These findings suggest this way of scoring the MB-CDI may be meaningful and useful in future analyses for distinguishing whether the effect of broadly specified “language” on behavior or in the brain is more closely tied to the learning of structure or predictors of sentence structure in a child’s language, or to their raw lexical inventory. This would be useful in studying disorders or individuals who may be developing across categories asymmetrically.

An important limitation of the study is the different sizes of content vs. function inventories, which is natural in any language. However, this means that almost all function words can be interrogated, whereas only a small fraction of content words can be asked about on a form of reasonable length, although the MB-CDI was carefully prepared to provide an accurate representation of common early English words. Furthermore, the model fit was marginal, suggesting that an instrument geared toward distinguishing function and content words could perform better. However, given the proliferation of studies using the MB-CDI, we propose the division proposed herein is still valuable. Finally, the measures remain highly correlated (FA: .79, one-weighted analysis; .87), making their effects difficult to distinguish.

The MB-CDI was designed for estimates of morphological and syntactic ability to come from the relevant sections. However, this paper shows that acquisition of function words, as reported by caregivers, is more closely related to structural ability than to acquisition of content words. We propose these factors as a way for investigating these separate abilities in future studies, especially when complete utterances or an MLU value are not available.

## Figures and Tables

**Table 1.** Demographic variables given in Wordbank for WG and WS. Percentages given as percent of non-missing data.

		WG		WS	
N		2435		5520	
Age in months (SD)		13.81 (2.34)		22.31 (4.71)	
Sex	Male	1235	48%	2105	49%
	Female	1157	52%	1989	51%
	Missing	43	-	1426	-
Ethnicity	Asian	46	4%	67	2%
	Black	124	12%	222	8%
	Hispanic	61	6%	131	5%
	White	774	73%	2202	81%
	Other	62	6%	96	3%
	Missing	1368	-	2805	-
Mother’s education	Primary	2	0%	8	0%
	Some secondary	69	6%	123	4%
	Secondary	254	24%	416	15%
	Some college	267	25%	613	22%
	College	294	27%	870	31%
	Some graduate	41	4%	162	6%
	Graduate	141	13%	584	21%
	Missing	1367	-	2744	-
Birth order	First	1429	51%	525	50%
	Second	931	34%	337	32%
	Third	291	10%	134	13%
	Fourth+	125	5%	51	5%
	Missing	2744	-	1388	-

**Table 2.** Descriptive statistics for the EFA/CFA divisions of the WG and WS forms. Percentages may not add to 100% because of rounding. For this analysis, mother’s education was converted to years and birth order simplified to first vs. later.

		WG		WS	
		EFA	CFA	EFA	CFA
N		1214	1422	2750	2770
Age in months (SD)		13.8 (2.3)	13.8 (2.4)	22.4 (4.7)	22.4 (4.7)
Sex	Male	47%	48%	38%	38%
	Female	51%	51%	36%	36%
	Missing	2%	2%	26%	26%
Ethnicity	Asian	2%	1%	1%	1%
	Black	5%	4%	4%	4%
	Hispanic	2%	2%	2%	2%
	White	33%	34%	39%	40%
	Other	2%	2%	2%	2%
	Missing	56%	56%	51%	51%
Mother’s education in years (SD)		14.6 (2.9)	14.7 (3.0)	15.6 (3.0)	15.6 (3.1)
Birth order	First	22%	21%	24%	24%
	Second+	21%	22%	26%	26%
	Missing	57%	57%	50%	50%

**Table 3.** The number of items in the categories shared and distinct between WG and WS. Where the categories are not named identically, the names are given as WG / WS. “Word Forms” is use of correct irregulars and “Word Endings” is use of incorrect overgeneralizations.

	Category	WG	WS
WG.I.D / WS.I.A	Sound Effects and Animal Sounds	12	12
	Animal Names / Animals	36	43
	Vehicles	9	14
	Toys	8	18
	Food and Drink	30	68
	Clothing	19	28
	Body Parts	20	27
	Small Household Items	36	50
	Furniture and Rooms	24	33
	Outside Things and Places to Go / Outside Things	27	31
	Places to Go		22
	People	11	29
	Games and Routines	19	25
	Action Words	55	103
	Descriptive Words	37	63
	Words about Time	8	12
	Pronouns	11	25
	Question Words	6	7
	Prepositions and Locations	11	26
	Quantifiers / Quantifiers and Articles	8	17
	Helping Verbs		21
	Connecting Words		6
WS.II	Word Forms (nouns/verbs)		5/20
	Word Endings (nouns/verbs)		14/31
	Complexity		37

**Table 4.** Factor loadings for the 2- and 3-factor EFAs on the Wordbank WS data. Loadings greater than 0.4 bolded “Word Forms” is use of correct irregulars and “Word Endings” is use of incorrect overgeneralizations. Categories with asterisks are easily considered function categories.

	Category	2-factor		3-factor		
		Lexical	Syntactic	Lexical	Functional	Morph.
Lexical (I.A)	Sound Effects and Animal Sounds	**0.92**	−0.20	**0.90**	−0.17	−0.03
	Animals	**1.00**	−0.06	**0.99**	−0.03	−0.01
	Vehicles	**1.02**	−0.10	**1.01**	−0.09	0.00
	Toys	**0.96**	0.01	**0.95**	0.01	0.02
	Food and Drink	**0.93**	0.06	**0.93**	0.04	0.04
	Clothing	**0.90**	0.09	**0.89**	0.09	0.02
	Body Parts	**1.04**	−0.12	**1.04**	−0.14	0.03
	Small Household Items	**0.90**	0.10	**0.90**	0.06	0.06
	Furniture and Rooms	**0.79**	0.21	**0.78**	0.20	0.04
	Outside Things	**0.86**	0.14	**0.84**	0.15	0.01
	Places to Go	**0.67**	0.33	**0.66**	0.33	0.03
	People	**0.64**	0.36	**0.63**	0.34	0.06
	Games and Routines	**0.91**	0.04	**0.90**	0.02	0.04
	Action Words	**0.68**	0.33	**0.66**	0.35	0.02
	Descriptive Words	**0.65**	0.37	**0.62**	**0.40**	0.00
	Words about Time	0.21	**0.74**	0.19	**0.75**	0.05
	* Pronouns	0.21	**0.77**	0.18	**0.79**	0.03
	* Question Words	0.16	**0.74**	0.13	**0.77**	0.03
	* Prepositions and Locations	0.39	**0.61**	0.36	**0.65**	0.00
	* Quantifiers and Articles	0.18	**0.79**	0.14	**0.84**	0.01
	* Helping Verbs	0.08	**0.87**	0.05	**0.91**	0.03
	* Connecting Words	−0.09	**0.91**	−0.13	**0.95**	0.03
Syntactic (II.B,C,E)	Word Forms, nouns	0.21	**0.54**	0.30	0.17	**0.43**
	Word Forms, verbs	−0.12	**0.98**	−0.04	**0.63**	**0.43**
	Word Endings, nouns	−0.02	**0.46**	0.11	−0.16	**0.77**
	Word Endings, verbs	−0.15	**0.67**	−0.05	0.10	**0.74**
	Complexity	0.03	**0.83**	0.10	**0.53**	0.36

**Table 5.** Factor loadings for the 2- and 3-factor EFAs on the Wordbank WG data. Loadings greater than 0.4 bolded. Categories with asterisks are easily considered function categories.

Category	2-factor		3-factor		
	1	2	1	2	3
Sound Effects and Animal Sounds	**0.94**	−0.22	−0.03	**0.85**	−0.06
Animal Names	**0.85**	0.08	**0.57**	**0.41**	−0.15
Vehicles	**0.75**	0.09	**0.74**	0.16	−0.27
Toys	**0.95**	−0.11	0.30	**0.62**	−0.14
Food and Drink	**0.78**	0.20	**0.65**	0.36	−0.11
Clothing	**0.68**	0.31	**0.81**	0.19	−0.13
Body Parts	**0.64**	0.33	**0.73**	0.24	−0.07
Furniture and Rooms	0.22	**0.72**	**1.06**	−0.20	0.03
Small Household Items	**0.52**	**0.49**	**0.91**	0.08	−0.05
Outside Things and Places to Go	**0.49**	**0.49**	**0.93**	0.03	−0.06
People	**0.74**	0.04	0.09	**0.72**	0.07
Games and Routines	**0.77**	0.12	0.09	**0.82**	0.14
Action Words	0.04	**0.92**	**0.87**	−0.04	0.28
Time Words	−0.17	**0.93**	**0.63**	−0.03	**0.40**
Descriptive Words	0.17	**0.81**	**0.76**	0.11	0.26
* Pronouns	0.25	**0.50**	0.18	**0.49**	0.37
* Question Words	−0.09	**0.69**	0.14	0.31	**0.54**
* Prepositions and Locations	0.37	**0.52**	**0.63**	0.20	0.12
* Quantifiers	0.13	**0.69**	**0.51**	0.21	0.31

**Table 6.** Model fit indices for WS CFAs. Typical criteria for “good fit” are given in the first row. CFI: comparative fit index; Tucker-Lewis index: TLI; RMSEA: root-mean-square error of approximation; SRMR: standardized root mean square residual. 95% confidence interval given for RMSEA. Chi-square tests are almost always significant (indicating bad fit) in large sample sizes.

Model	Chi-square *p*	Robust CFI	Robust TLI	RMSEA	SRMR
*Criteria*	*>.05*	*⩾0.9*	*⩾.9*	*<.08*	*<.08*
WS 2-factor	<.001	.935	.927	.097 [.095, .099]	.041
WS 3-factor	<.001	.944	.942	.101 [.098, .104]	.038

## References

[R1] Bates E, & Goodman JC (1999). On the emergence of grammar from the lexicon. In MacWhinney B (Ed.), The emergence of language. Lawrence Erlbaum.

[R2] Bates E, Marchman V, Thal D, Fenson L, Dale P, Reznick JS, Reilly J, & Hartung J (1994). Developmental and stylistic variation in the composition of early vocabulary. Journal of Child Language, 21(1), 85–123. 10.1017/S0305000900008680 8006096

[R3] Bauer DJ (2017). A more general model for testing measurement invariance and differential item functioning. Psychological Methods, 22(3), 507–526. 10.1037/met0000077 27266798 PMC5140785

[R4] Berglund E, Eriksson M, & Westerlund M (2005). Communicative skills in relation to gender, birth order, childcare and socioeconomic status in 18-month-old children. Scandinavian Journal of Psychology, 46(6), 485–491. 10.1111/j.1467-9450.2005.00480.x 16277649

[R5] Bhat DNS (2000). Word classes and sentential functions. In Vogel PM & Comrie B (Eds.), Approaches to the typology of word classes. Mouton de Gruyter.

[R6] Bouchard C, Trudeau N, Sutton A, Boudreault M-C, & Deneault J (2009). Gender differences in language development in French Canadian children between 8 and 30 months of age. Applied Psycholinguistics, 30(4), 685–707. 10.1017/S0142716409990075

[R7] Brown R (1973). A first language: The early stages. Harvard U. Press.

[R8] Brown TA (2015). Confirmatory Factor Analysis for Applied Research, Second Edition. Guilford Publications. http://ebookcentral.proquest.com/lib/umn/detail.action?docID=1768752

[R9] Carroll JB (1957). Biquartimin criterion for rotation to oblique simple structure in factor analysis. Science, 126, 1114–1115. 10.1126/science.126.3283.1114 17755543

[R10] Christophe A, Millotte S, Bernal S, & Lidz J (2008). Bootstrapping Lexical and Syntactic Acquisition. Language and Speech, 51(1–2), 61–75. 18561544 10.1177/00238309080510010501

[R11] Corver N, & van Riemsdijk H (2013). Semi-lexical categories. In Corver N & van Riemsdijk H (Eds.), Semi-lexical categories: The function of content words and the content of function words (2nd ed.). De Gruyter Mouton.

[R12] Dixon RMW & Aikhenvald A (2004). Adjective Classes: A Cross-Linguistic Typology. Oxford University Press.

[R13] Dromi E (1987). Early lexical development. Cambridge University Press.

[R14] Fenson L, Marchman VA, Thal DJ, Dale PS, Reznik JS, & Bates E (2007). MacArthur-Bates Communicative Development Inventories: User’s Guide and Technical Manual (2nd ed.). Brookes Publishing Co.

[R15] Frank MC, Braginsky M, Yurovsky D, & Marchman VA (2017). Wordbank: An open repository for developmental vocabulary data. Journal of Child Language, 44(3), 677–694. 10.1017/S0305000916000209 27189114

[R16] Garnier S (2018). viridis: Default Color Maps from “matplotlib” (0.5.1) [Computer software]. https://CRAN.R-project.org/package=viridis

[R17] Gleason JB, & Ratner NB (2017). The Development of Language (9th ed.). Pearson.

[R18] Hoff-Ginsberg E (1998). The relation of birth order and socioeconomic status to children’s language experience and language development. Applied Psycholinguistics, 19(4), 603–629. 10.1017/S0142716400010389

[R19] Hooper D, Coughlan J, & Mullen MR (2008). Structural Equation Modelling: Guidelines for Determining Model Fit. The Electronic Journal of Business Research Methods, 6(1), 53–60.

[R20] Hyams N, & Orfitelli R (2015). The Acquisition of Syntax. In Cairns H & Fernandez E (Eds.), Handbook of Psycholinguistics (p. 19). Wiley/Blackwell Publishers.

[R21] Kuhl PK, Conboy BT, Padden D, Nelson T, & Pruitt J (2005). Early Speech Perception and Later Language Development: Implications for the “Critical Period”. Language Learning and Development, 1 (3–4), 237–264. 10.1080/15475441.2005.9671948

[R22] Kuhl PK, Stevens E, Hayashi A, Deguchi T, Kiritani S, & Iverson P (2006). Infants show a facilitation effect for native language phonetic perception between 6 and 12 months. Developmental Science, 9(2), F13–F21. 10.1111/j.1467-7687.2006.00468.x 16472309

[R23] Kuhl PK, Tsao F-M, & Liu H-M (2003). Foreign-language experience in infancy: Effects of short-term exposure and social interaction on phonetic learning. Proceedings of the National Academy of Sciences, 100(15), 9096–9101. 10.1073/pnas.1532872100 PMC166444 12861072

[R24] Moon C, Lagercrantz H, & Kuhl PK (2013). Language experienced in utero affects vowel perception after birth: A two-country study. Acta Paediatrica (Oslo, Norway : 1992), 102(2), 156–160. 10.1111/apa.12098 23173548 PMC3543479

[R25] Pancsofar N, & Vernon-Feagans L (2006). Mother and father language input to young children: Contributions to later language development. Journal of Applied Developmental Psychology, 27(6), 571–587. 10.1016/j.appdev.2006.08.003

[R26] R Core Team. (2020). R: A Language and Environment for Statistical Computing (4.0.0) [Computer software]. R Foundation for Statistical Computing. https://www.R-project.org

[R27] Revelle W (2018). psych: Procedures for Psychological, Psychometric, and Personality Research (R package version 1.8.12) [Computer software]. Northwestern University. https://CRAN.R-project.org/package=psych

[R28] Rosseel Y (2012). lavaan: An R Package for Structural Equation Modeling. Journal of Statistical Software, 48(1), 1–36. 10.18637/jss.v048.i02

[R29] Valian V (1986). Syntactic categories in the speech of young children. Developmental Psychology, 22(4), 562. 10.1037/0012-1649.22.4.562

[R30] Van Hulle CA, Goldsmith HH, & Lemery KS (2004). Genetic, Environmental, and Gender Effects on Individual Differences in Toddler Expressive Language. Journal of Speech, Language, and Hearing Research, 47(4), 904–912. 10.1044/1092-4388(2004/067) 15324294

[R31] Werker JF, & Yeung HH (2005). Infant speech perception bootstraps word learning. Trends in Cognitive Sciences, 9(11), 519–527. 10.1016/j.tics.2005.09.003 16202639

[R32] Wickham H, Averick M, Bryan J, Chang W, McGowan L, François R, Grolemund G, Hayes A, Henry L, Hester J, Kuhn M, Pedersen T, Miller E, Bache S, Müller K, Ooms J, Robinson D, Seidel D, Spinu V, … Yutani H (2019). Welcome to the Tidyverse. Journal of Open Source Software, 4(43), 1686. 10.21105/joss.01686

[R33] Woods CM, & Edwards MC (2011). Factor Analysis and Related Methods. In Rao CR, Miller JP, & Rao DC (Eds.), Essential Statistical Methods for Medical Statistics (pp. 174–201). North-Holland. 10.1016/B978-0-444-53737-9.50009-8

